# Multi-Modal Data Analysis for Pneumonia Status Prediction Using Deep Learning (MDA-PSP)

**DOI:** 10.3390/diagnostics12071706

**Published:** 2022-07-13

**Authors:** Ruey-Kai Sheu, Lun-Chi Chen, Chieh-Liang Wu, Mayuresh Sunil Pardeshi, Kai-Chih Pai, Chien-Chung Huang, Chia-Yu Chen, Wei-Cheng Chen

**Affiliations:** 1 Department of Computer Science, Tunghai University, Taichung 407224, Taiwan; rickysheu@thu.edu.tw (R.-K.S.); kcpai@thu.edu.tw (K.-C.P.); ccwhung@vghtc.gov.tw (C.-C.H.); g08350007@thu.edu.tw (C.-Y.C.); g10350003@thu.edu.tw (W.-C.C.); 2 Department of Critical Care Medicine, Taichung Veterans General Hospital, Taichung 40705, Taiwan; clwu@vghtc.gov.tw; 3 Department of Industrial Engineering and Enterprise Information, Tunghai University, Taichung 407224, Taiwan; 4 Department of Automatic Control Engineering, Feng Chia University, Taichung 407102, Taiwan; 5 AI Center, Tunghai University, Taichung 407224, Taiwan; mayuresh@thu.edu.tw

**Keywords:** multi-modal data, status prediction, interpretable AI, pulmonary respiratory disease

## Abstract

Evaluating several vital signs and chest X-ray (CXR) reports regularly to determine the recovery of the pneumonia patients at general wards is a challenge for doctors. A recent study shows the identification of pneumonia by the history of symptoms and signs including vital signs, CXR, and other clinical parameters, but they lack predicting the recovery status after starting treatment. The goal of this paper is to provide a pneumonia status prediction system for the early affected patient’s discharge from the hospital within 7 days or late discharge more than 7 days. This paper aims to design a multimodal data analysis for pneumonia status prediction using deep learning classification (MDA-PSP). We have developed a system that takes an input of vital signs and CXR images of the affected patient with pneumonia from admission day 1 to day 3. The deep learning then classifies the health status improvement or deterioration for predicting the possible discharge state. Therefore, the scope is to provide a highly accurate prediction of the pneumonia recovery on the 7th day after 3-day treatment by the SHAP (SHapley Additive exPlanation), imputation, adaptive imputation-based preprocessing of the vital signs, and CXR image feature extraction using deep learning based on dense layers-batch normalization (BN) with class weights for the first 7 days’ general ward patient in MDA-PSP. A total of 3972 patients with pneumonia were enrolled by de-identification with an adult age of 71 mean ± 17 sd and 64% of them were male. After analyzing the data behavior, appropriate improvement measures are taken by data preprocessing and feature vectorization algorithm. The deep learning method of Dense-BN with SHAP features has an accuracy of 0.77 for vital signs, 0.92 for CXR, and 0.75 for the combined model with class weights. The MDA-PSP hybrid method-based experiments are proven to demonstrate higher prediction accuracy of 0.75 for pneumonia patient status. Henceforth, the hybrid methods of machine and deep learning for pneumonia patient discharge are concluded to be a better approach.

## 1. Introduction

Pneumonia was ranked the third cause of death in Taiwan and the mortality rate was 64.4/100,000 in 2019 [1]. The old age (>65 y/o) and severity of pneumonia were associated with higher mortality and longer hospital stays [2,3,4,5]. According to Taiwan’s guidelines, community-acquired pneumonia (CAP) is defined as a pulmonary parenchymal acute infection in patients who acquire the condition in the community [6]. The Taiwan guideline suggested treating CAP by empirical, the severity of pneumonia (CURB-65), and risk of multidrug-resistant organisms even if the microbiologic diagnosis was done [6]. However, even with the empirical antibiotics used, the clinical response rate was 72.7% [7], intensive care unit (ICU) admission rate increased from 9.5 to 20%, ventilation rate was 10.1–20.1%, and length of ICU stay was 19.9 ± 22.8 days according to age [3,7,8]. The American Thoracic Society (ATS) and Infectious Diseases Society of America (IDSA) suggest criteria to define when a hospitalized patient with CAP has reached clinical stability [9,10]. Early clinical stability offers information about antibiotic treatment (e.g., the appropriateness of such treatment, switching to oral medication, and short antibiotic treatment durations) and indications for hospital discharge that reduce hospital length-of-stay [11,12,13,14]. Moreover, early clinical response is another predictor with lower mortality [11,15,16,17,18,19,20], lower length of stay [11,16,17,18,19], lower readmission due to pneumonia [15,20], lower intensive care unit (ICU) admission [11,16,18], lower intubation rate [11], lower acute kidney injury (AKI) and need of renal replacement [11], lower 30-day readmission [16], and lower respiratory failure [18]. The earlier time to clinical response means a lower length of stay and lower cost [20]. Whereas the research work [11] showed early responders (≤3 days) vs. late responders had a better outcome, including lower mortality, lower length of stay, making lower costs and improving therapy quality. Many papers discussed the predictor of early clinical response or early time to clinical stability [11,15,16,17,18,19,20,21,22]. The risk factors associated with late clinical response or late time to clinical stability includes more comorbidity [11,16,18], more severity of disease (either PSI or CURB-65) [11,18,19], old age [16,19], fewer sign/symptoms [16], less respiratory compromise [16], lower platelet count [16], GNB pneumonia [17,19], altered mental status [17], lower arterial pH [17], with heart failure [17], liver disease [18], more pleural effusion [18], multilobar CAP [18,19], and legionella pneumonia [19].

Pneumonia is one of the main causes of mortality in all ages of the population, especially in elderly patients [20]. To avoid pneumonia, the flu vaccine is required to be administered every year about which case the mass population is not familiar enough. Nevertheless, wearing a mask, healthy lifestyle, good hygiene, and avoiding smoking are some of the precaution measures that can be practiced to avoid pneumonia. Several clinical factors are available about the patients during checkups and treatment in the hospital, i.e., vital signs, CXR, computational tomography (CT) scan, etc. The assigned/treating doctors usually check the record report of the patient at a specific time interval to confirm the status of pneumonia after starting treatment. To address this problem, a need for artificial intelligence (AI) is felt to detect the treatment response of pneumonia within the affected patients [20,21]. Therefore, this paper demonstrates a multi-modal data analysis for pneumonia status prediction (MDA-PSP) as an AI-based process. To implement the system, we have studied the working process of in-charged physicians. The features included are age, gender, vital signs, CXR, and laboratory data, which are challenging to collect and have many missing values. Thus, to improve the treatment process, MDA-PSP will first analyze the patient’s vital signs report and then check for CXR, which will provide a combined automated evaluation for the high accuracy prediction of the patient’s pneumonia status. The designed AI system model will simulate the doctor’s decision-making process, establish a predictive model, and predict on the third day of hospitalization whether the patient can be discharged on the seventh day, while improving the quality of medical care and reducing medical expenses. The doctors are thus able to perform optimal management for those pneumonia patients with short (less than or equal to 7 days) and long (more than 7 days) hospital stays. Moreover, high precision of patient discharge prediction will increase the utilization of hospital resources including the ward, bed, services, etc.

The clinical stability of community-acquired pneumonia (CAP) treatment can be achieved by using early response to treatment [11]. The first 72 h are set to be a treatment endpoint by the US FDA for a clinical trial. Considering the evaluations demonstrated by several studies [20,21], they are unaware to determine whether the patient can be discharged from the hospital or not on the 7th day of admittance.

Several experiments show that different disease parameters and CNN models are used for pneumonia prediction, but they lack complete analysis based on hospital standards including vital signs and CXR analysis. Therefore, a need to provide a complete hospital treatment for the process of predicting discharge status of patients, with MDA-PSP consisting of a hybrid approach having vital signs and CXR analysis using deep learning-based classification. Table 1 shows the different pneumonia prediction comparisons in detail.

### 1.1. MDA-PSP Objectives

- Scrutinizing the digital data for the pneumonia prediction using artificial intelligence: Artificial intelligence is considered to be an important factor for the pneumonia prediction in patients. Therefore, the MDA-PSP uses combined vital signs and CXR of patients to predict pneumonia status using a trained model and classifying them using deep learning.
- Leverage medical doctor’s experience for the pneumonia status: MDA-PSP provides an effective solution for pneumonia prediction based on different parameters of 19 vital signs and five different sections in CXR multi-modal data analysis with their combinations. Thus, combining the patient’s physiological data and chest X-ray image’s within first 3 days and the training model to achieve better accuracy by the predictive model interpretability benefits the physicians analysis.
- Mean time analysis of the patient’s pneumonia recovery within the next 7 days: The analysis is evaluated by a trained model consisting of SHAP and dense layers with class weights having the input readings from the initial to 2nd/3rd day of vital signs and CXR that can effectively predict the recovery status of the patient on the 7th day.

### 1.2. Literature Survey

**Figure 1:**
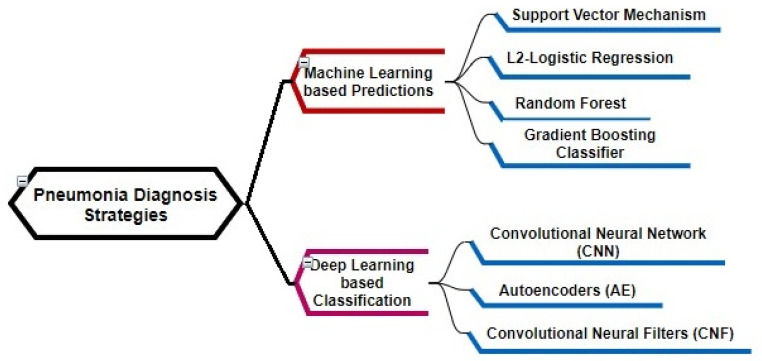
Pneumonia Prediction Strategy Analysis.

Recently, the use of artificial intelligence has been found to be beneficial within medical hospitals for improving the quality of disease diagnosis, recovery prediction, and decreasing the analysis related errors in vital signs as well as radiology. At present, there is no combination of clinical data and CXR data to establish a machine learning model to simulate doctors’ decision-making to assist in the care of community-type pneumonia patients. Therefore, this paper approaches the MDA-PSP multi-modal data analysis to achieve high accuracy for the pneumonia prediction process by using vital signs and CXR images using pre-processing and classification, respectively. The recent studies structure shown in Figure 1 is used to define the pneumonia symptoms-based prediction analysis, which include machine learning, deep learning classifiers, and hybrid models.

Several machine learning (ML) based models for pneumonia prediction include support vector mechanism (SVM), L2-logitic regression, random forest, gradient boosted classifier (GBC), XGBoost, etc. Acute respiratory disease syndrome (ARDS) detection by label uncertainty accounting using machine learning is presented by N. Reamaroon et al. [26]. Grading by confidence weights is assigned to labels for training input to SVM for good confidence in prediction. Additionally, a time-series method analyses for patient’s clinical data inter-correlation by limiting overfitting is an improvement over classical SVM.

Stratification of patient risk for ARDS using machine learning is demonstrated by D. Zeiberg et al. [22]. The model uses EHR as features input to L2-logitic regression, successfully identifying patients observed for at least the first 6 h with hypoxia. Moreover, the detail model validation with multi-fold cross validation with optimal hyper-parameter tuning is performed. A machine learning predictive model for ARDS event analysis in ICU patients is demonstrated by X.-F. Ding et al. [27]. A random forest model is used with parameters including the decision tree numbers and random subset feature size. Eventually, a best split and k feature selection approach provides a better ROC curve. Diagnosis of ICU patients with their ventilation management by using machine learning and IoT is presented by G. Rehm et al. [28]. The data used is only of approximately 35 patients with k-fold cross validation, synthetic minority oversampling technique (SMOTE) for extremely random trees classifier (ERTC), GBC, and multi-layer perceptron (MLP) as ML algorithms. The data processing is done as micro-batch processing on the Amazon Web Services (AWS) cloud platform of 5 min sampling. ARDS identification from readily available clinical data by ML classifier models is demonstrated by P. Sinha et al. [29]. The dataset includes demographic, respiratory-based parameters, vital signs, and laboratory data to be processed by the 10-fold cross validation, which is evaluated by a gradient boosting machine (GBM) and XGBoost ML models in R. The evaluation is based on mortality at day 90 in different phenotypes by applying classifiers. The early prediction of ARDS using supervised ML is presented by S. Le et al. [30]. The MIMIC-III dataset is used similarly to the previous reference approach of 10-fold cross validation and XGBoost for early prediction of ARDS. The evaluation is performed on parameters of age, ICU ward admission, MEWS severity patients at admission, and median length-of-stay at 12 h, 24 h, and 48 h demographics for 1.90 k patients yielding good prediction. Some hybrid methods of machine learning, artificial neural network, genetic algorithm, and natural language are found to be also effective in ARDS [31,32,33,34,35,36].

For CXR, symptom detection possesses multiple image processing and computer vision techniques that are discussed below as CNN, auto-encoder (AE), convolutional neural filter (CNF), etc. Chest X-rays (CheXNet) source-based pneumonia detection by radiology level analysis with deep learning is demonstrated by P. Rajpurkar et al. [37]. A convolutional model of 121 layers is designed to detect 14 different types of diseases by using heat maps, class activation mappings (CAMs), and feature maps. One hundred thousand frontal X-rays are used for analysis and detection resulting in higher F1 score. CXR images used for rib suppression by ICA method is presented by HX Nguyen et al. [38]. The use of rib suppression helps in early lung nodule detection by histogram equalization and frangi filter with independent component analysis (ICA). The Japanese Society of Radiological Technology (JSRT) database is used with images of 154 nodules and 93 non nodules having high resolution beneficial for training and testing. Bone suppression in chest radiography by deep learning is demonstrated by M. Gusarev et al. [39]. A stacked denoising autoencoder (AE) is used with a multi-layer convolutional neural model with reduced mean squared error (MSE) and maximized multi-scale structural similarity (MS-SSIM). An educational dataset is used with contrast-limited adaptive histogram equalization (CLAHE) for local contrast enhancement. Effectively performing bone suppression by CNF in chest X-ray is presented by N. Matsubara et al. [40]. Overlapping of bones in the lung fields can be affected by bone suppression using CNF based on CNN having a spatial filter configured by convolution, max pool, and fully connected layers. The dataset uses CT volume of cancer imaging by the National Institute of Health (JSRT), claiming higher experiments accuracy without losing soft-tissue information. Recently, several researchers used CXR as a medium to detect respiratory infection by deep learning in many studies [41,42,43,44]. The plan to organize and present research contents can be stated as Section 3 materials and methods containing architecture, its functioning, system model, algorithms, flowcharts and their respective description. In Section 4, results of the system configuration, dataset details, experiments, and results are presented. Finally, Section 5 has a discussion followed by conclusion for the research, acknowledgments, references, and Appendix.

## 2. Materials and Methods

Hospital Research Project and Approach: The MDA-PSP project is performed in Taichung Veterans General Hospital (TCVGH), which consists of a total 1500 beds for the patients in the central region of Taiwan.

### 2.1. Hospitalization Conditions and Criteria

#### 2.1.1. Pre-Requisite and Hospitalization Criteria of the Patients

This work consists of patients with the criteria of admittance of the patients in the general ward. The patient should be an adult with an age 18 or higher and diagnosed with community acquired pneumonia < 48 h after admission. Whereas the patients excluded are due to the criteria of having one or multiple of the following: (a) A patient is directly admitted in the ICU, (b) The patients who have died in the hospital, (c) The patient is admitted for less than 3 days, (d) Acute respiratory failure and in need of a ventilator, (e) Admission to other hospital >72 h due to pneumonia, and (f) Women who are pregnant or need to provide breast-feeding. The vital-sign dataset given as input to the model is presented in the form of detailed statistics in Table 2. The dataset consists of various parameters, which have a specific range and is calculated for 3 consecutive days within the hospital. Every patient’s data is presented in the form of mean, median, and mode values for the detailed statistics of day 1, day 2, and day 3.

**Figure 2:**
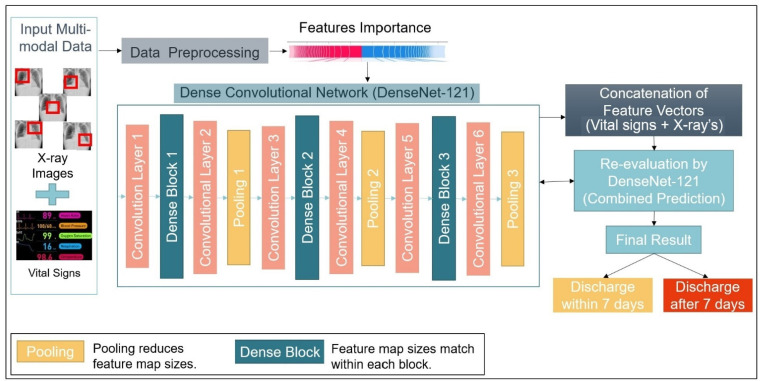
MDA-PSP System Model.

Table 3 presents the comorbidity data details, which is categorized as per the patient’s affected by the disease and the percentage of the same patients discharged within 7 days and after 7 days. Table 4 summarizes the various vital signs with their respective categories. All the vital signs within the dataset are categorized as lab data, comorbidity data, basic vital signs, and scores based on the 3 days’ patients collected data. The comorbidity data shows the scope of complications that may affect the discharge of the patient’s status, which can be more or less severe. Table 5 presents the vital signs checked by the physician as the basic factor for any treatment and its overcoming analysis. All the patients are adults, so the age is considered only for those greater than or equal to 20 years. The pulse rate also known as heart rate determines the number of times the heart beats per minute. The amount of oxygen traveling through a human body with his/her red blood cells is known as oxygen saturation or “O2 sats”. The healthy adult’s oxygen saturation is usually found within the range 95% to 100%. The respiratory rate is the breaths taken by the adult per minute. Figure 2 presents the MDA-PSP system model, which can be explained in four parts as multi-modal input data, data pre-processing with feature importance, dense layer architecture, and evaluation of the final results.

#### 2.1.2. The Decision for Discharge within 7 Days

The MDA-PSP is designed to predict the status of the pneumonia infection. As all the patients considered are affected by pneumonia, the prediction of the pneumonia status after the treatment is made available. Usually, the patients with pneumonia are given one course of antibiotics for 5 to 7 days. The training data consists of outcome after the 3 days of the patient’s status which is based on the doctor’s decision. Multiple factors involved in the doctor’s decision for discharge are the X-ray status, vital sign status, and the basic vital signs given in Table 5. The prediction determines whether the patient is discharged within (less than or equal to) 7 days or more than 7 days. Eventually, if the overall prediction is worse, then the discharge is given probably after 7 days. Whereas if the prediction is improving, then discharge is given within 7 days to the patient.

#### 2.1.3. Input Data for Multi-Modal Analysis

The input given to this system is CXR images and vital signs data as shown on the left most section of Figure 2 for the multi-modal data analysis. Every CXR image has 5 sections of top-left, bottom-left, top-right, bottom-right, and center. The first four sections are used to check infiltrates, whereas the center section is used to check for cardiomegaly. In the case of vital sign data, the details are presented by the identifiers/labels and statistics as shown in Table 2.

### 2.2. Data Preprocessing

To achieve accurate attribution values and consistency, SHAP preprocessing is utilized for balanced weights distribution in the feature model [45]. The additive feature attribution methods possess local accuracy, missingness, and consistency. The feature attribution sum is equal to the function output stated as local accuracy. A missing feature is credited with no importance and is given as missingness (zi’ = 0). Whereas a large impact feature is obtained even after the model change and has no decrease in the attribution, which is known as consistency. The attribution method for additive features is known to have g as an explanation model, which uses binary variable linear function:

(1) $$g(z^{\prime })=\emptyset_{0}+\sum_{i=1}^{M}\emptyset_{i}z_{i}^{\prime }$$

Here, the $z^{\prime }\epsilon {\left\{0,1\right\}}^{M},$ $\emptyset_{i}\epsilon R,$ and *M* is the number of input features. The observed feature is usually represented by ($z_{i}^{;}=1$) or by ($z_{i}^{;}=0$) as unknown and the attribution values for features is given as $\emptyset_{i}$’s. There are multiple important techniques for data pre-processing that include data cleaning, data rule definition, and data supplement.

**Figure 3:**
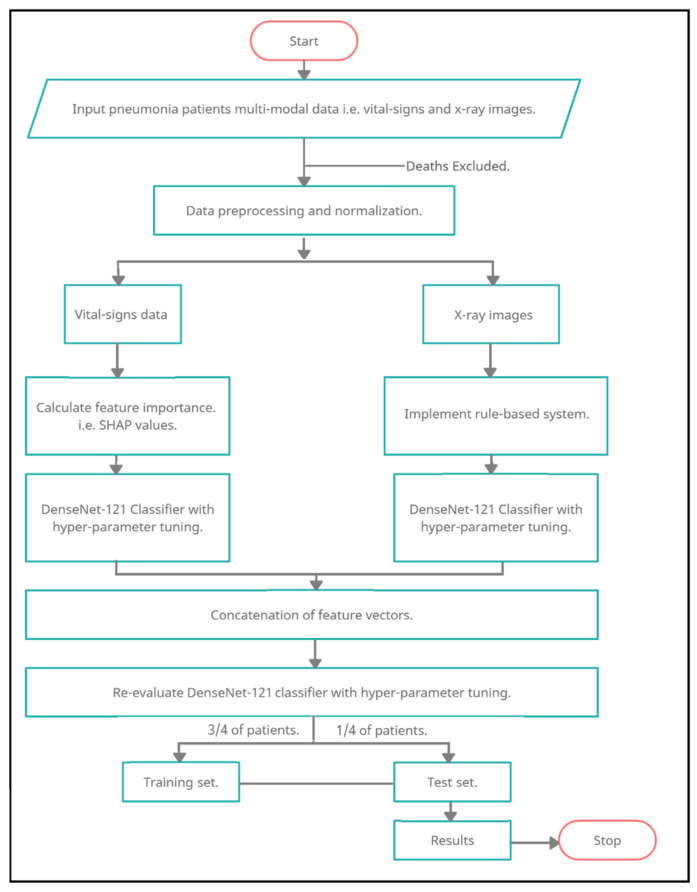
MDA-PSP Data Flow Diagram.

An MDA-PSP data flow diagram presents the plan and implementation details as shown in Figure 3. At the start, the MDA-PSP system takes the input of multi-modal data in the form of several patient’s CXR and vital signs data. Initially, all the patients who are affected by pneumonia and recovered are only considered, whereas the patients who have died during the arrival/treatment/multiple disease disorder are excluded from this work. In the data pre-processing stage, the data is normalized and standardized to avoid any miscalculations in the predictions. A separate algorithm is applied for the processing of CXR and vital signs, so as to provide the best results in their respective category. In the case of vital signs data, a feature importance is first analyzed to capture the top set of features for better predictions by using SHAP. The SHAP features are then given as input to the dense layer classifier with hyper-parameter tuning [46,47] for best parameter orchestration. In a similar way, the CXR images are processed by using the gray scale conversion, resized to a standard values, and inference rules-based system is approved by the medical doctors. Later, a dense layer classifier with hyper-parameter tuning is implemented.

Successively, the concatenation of feature vectors from both dense layers of multimodal data is combined and re-evaluated with dense layers having new hyper-parameters consisting of ⅔ and ⅓ ratios for training and testing, respectively.

### 2.3. Functional Model

**Figure 4:**
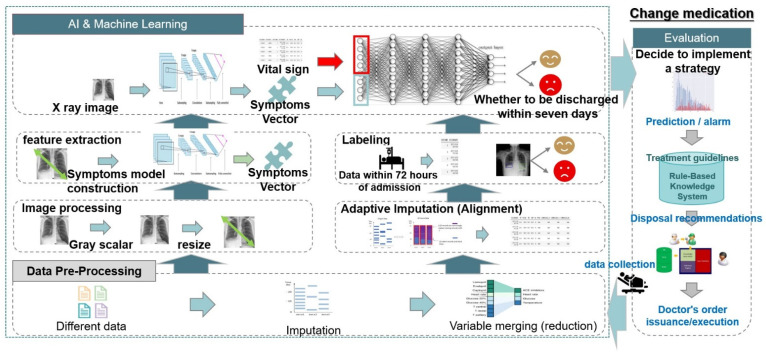
The MDA-PSP Functional Model.

The detailed implementation of the MDA-PSP is shown in the form of a functional model in Figure 4. In the beginning, the doctor initiates the process by providing the input data to the MDA-PSP system. The vital sign data is then preprocessed by using the imputation, i.e., substitute missing values by using series mean, median, average, etc. The variables are then reduced by using categorical grouping. The input multi-modal data is then improved using the hospital’s standard operating procedure (SOP)-based adaptive imputation, i.e., advance data pre-processing of univariate for statistical, multi-variate for regressions, and time series/interpolation for algebraic operations. The labeling of the raw data first needs to be performed by the domain experts/medical doctors to possess high quality training data. The labeling of data is performed on the trained first 3 days (72 h) of the patients records after the treatment that can be evaluated by the outcome for 0 (No Discharge) or 1 (Discharge) to the patient.

Similarly, the image CXR data then can be pre-processed by using computer vision operations on the greyscale, resize, etc. Later, the symptom model construction is performed by the DNN algorithm by producing a symptom vector as the output. Ultimately, the final hybrid model of DNN-BN, which takes a combined symptom vector from the vital signs and CXR data, is then evaluated to predict the outcome as either 0 or 1. This outcome is then utilized by the medical doctors by applying inference rule-based knowledge systems. It is specified as checking the outcome, age, pulse rate, SaO2, respiratory rate, and comorbidity disease stage. Therefore, the decision is then given for the final discharge to the patient.

### 2.4. Algorithms

This subsection is used to present the various MDA-PSP algorithms with pseudo-code for the detail working. The pre-processing is performed by using the imputation and adaptive imputation for the patient’s vital signs data. Basically, the purpose of pre-processing is avoiding inconsistency within the dataset and its limitations. The experiments section demonstrates the results obtained by using this method.

Algorithm 1 given above is used to present the data preprocessing performed on the patient’s vital signs data, CXR images, and has obtained feature vectors from it. Later, the feature vectors of the vital sign data and CXR images are used as the multi-modal data analysis, which is used to predict the pneumonia status of the patient. In step 1, vital signs record of the patient is taken as input in the form of raw data (*DRaw, IRaw*). In step 2, the vital sign data and CXR images are being labeled by the doctors for the exceptional cases in the combined training and test set. In step 3, the inference rules (*RInference*) have the conditions specified by the doctors. In step 4, the threshold determines the cut off limit for the decision for the patient’s discharge. In step 5, the output is given in the form of NN confidence (*NN_Conf_*) and in step 6, the MDA-PSP system alerts the prediction of the patient’s discharge within the 7 days or not. In step 7, the candidate set 1, candidate set 2, the *NN_Conf_*, and the candidate final (*candidate_Final_*) used for storing the feature vectors are initialized to NULL. In step 8, the vital sign raw data (*DRaw*) is pre-processed by using the imputation (*DProcessed_IM_*) by the mean, median, or by k-nearest neighbor algorithm. The preprocessing is performed on the input (*IRaw*) by the imputation and stored in the CXR image processed (*IProcessed_IM_*). Later in step 9, the *DProcessed_IM_* is used to map the multiple data by reducing it to a single category (*DProcessed_CG_*) for identification. The CXR image (*IProcessed_IM_*) is divided into four sections as the upper-left, lower-left, upper-right, and lower-right to capture the various patterns for the pneumonia by identifying the infected area and is stored as a categorized image (*IProcessed_CG_*). In step 10, the vital signs preprocessed data (*DProcessed_VS_*) is obtained by combining the adaptive imputation data for univariate, multivariate, and time series/interpolation with the labeling performed by the medical doctor/domain expert (*DProcessed_CG_*). In step 11, the CXR image is set to standard size and converted to gray scale for better image processing, which is later labeled by the doctor to provide detailed identification patterns (*IProcessed_IP_*) on the current dataset. In step 12, the inference rules (*RInference*) are set to be checked for preliminary conditions including the patient affected by pneumonia and admitted in the general ward of the hospital. Whereas the clinical checkup parameters as specified in Table 5 includes age, pulse rate, oxygenation (SaO2), respiratory rate and comorbidity for multiple diseases with each consisting of 20% points are rated based on doctor’s observations. In step 13, if the *DProcessed_VS_* is found to be consistent and if the score counted from the inference rules (*RInference*) is greater than or equal to that specified by the doctor, which is considered to be ready to be processed further, then in step 14, by using the dense layer, the feature vectors are captured from the *DProcessed_VS,_* and the processed image (*IProcessed_IP_*) and is stored in candidate Set 1 and candidate Set 2, respectively, instep 15. In step 16, the data is considered to be inconsistent or the score is less than the threshold, then an error message is printed by the system in step 17 as inconsistent data. In step 18, as the inconsistent data is found to be insufficient for processing, then the algorithm terminates. In step 19, the candidate finally stores the combined feature vectors of the vital signs and CXR image as the candidate Set 1 and candidate Set 2, respectively. In step 20, the NN confidence (*NN_Conf_*) is the dense layer with batch normalization (BN) prediction for the input with candidate final (*candidate_Final_*). In step 21, if the condition checks whether NN confidence (*NN_Conf_*) is greater than or equal to the threshold, then the MDA-PSP system raises an alert to allow discharge for the recovered patient in step 22. Training this model will help doctors determine whether a patient is discharged from the hospital within 7 days. In addition to strengthening treatment, the mobility of the bed becomes higher and medical resources can be used more effectively. In step 23, if NN confidence (*NN_Conf_*) is less than the threshold value then an alert is raised for no discharge to the patient as the recovery is not satisfactory in step 24. Finally, in step 25, the NN confidence (*NN_Conf_*) and alert is returned by the system.
**Algorithm 1:** Pneumonia Status Prediction by Data Preprocessing and Feature Vectorization using Multi-Modal Data AnalysisInput:  *DRaw and IRaw*, Vital signs record and CXR images of the patients.     *DLabelled and ILabelled_,_* Labelling performed by the doctors.     *RInference_,_* Inference rules with threshold specified by the doctors.     *Threshold*, Determines pneumonia status prediction limit.Output:  *NN_Conf_*, NN model predicted confidence.      *Alert*, MDA-PSP system alert for discharge or no discharge within 7 days.Initialize (*candidateSet* 1, *candidateSet* 2, *NN_Conf_*, *Candidate_Final_*) *=* ∅*DProcessed_IM_, IProcessed_IM_* = Imputation (*DRaw, IRaw*)*DProcessed_CG_, IProcessed_CG_* = Categorization (*DProcessed_IM_, IProcessed_IM_*)*DProcessed_VS_* = Adaptive Imputation (*DProcessed_CG_*) ⋃ *DLabelled**IProcessed_IP_* = Grey-Scale (Resize (*IProcessed_CG_*)) ⋃ *ILabelled**RInference =* Pneumonia ˄ General Ward ˄ Clinical Checkup ˄ ComorbidityIf *DProcessed_VS_* is Consistent and *Score (RInference) ≥ Threshold* then   Feature Vectors_VS_, Feature Vectors_IP_ = CNN (*DProcessed_VS_, IProcessed_IP_)*     *candidate Set 1, candidate Set 2 =* Feature Vectors_VS_, Feature Vectors_IP_else   Print “Inconsistent Data”   break*candidate_Final_* = *candidate Set* 1 ⋃ *candidate Set* 2*NN_Conf_ = Dense-BN(Candidate_Final_)*If *NN_Conf_* ≥ Threshold then   Alert “Discharge”else   Alert “No Discharge”Return *NN_Conf_*, *Alert*

## 3. Results

In this section, all the experiments carried out for the model implementation, comparisons, and results will be presented. The experiments and testing performed on the hardware for this model is presented in Table 6. A series of experiments will be presented in the following subsections that will be evaluated by using a detailed analysis and its respective implementation. In this work, we simulate the doctor’s decision-making process, establish a predictive model, and predict whether a patient can be discharged on the seventh day by the third day of hospitalization. Ultimately, this may improve the quality of medical care and reduce medical expenses. The focus here will be to understand the behavior of data with different preprocessing and its suitable machine and deep learning methods to achieve a better combination for the evaluations. Nevertheless, the problem needs to be clearly defined as the incorrect information makes the experiment invalid.

### 3.1. Data Preprocessing

#### 3.1.1. Vital Signs for Data Observations, Feature Scoring and Evaluation

**Figure 5:**
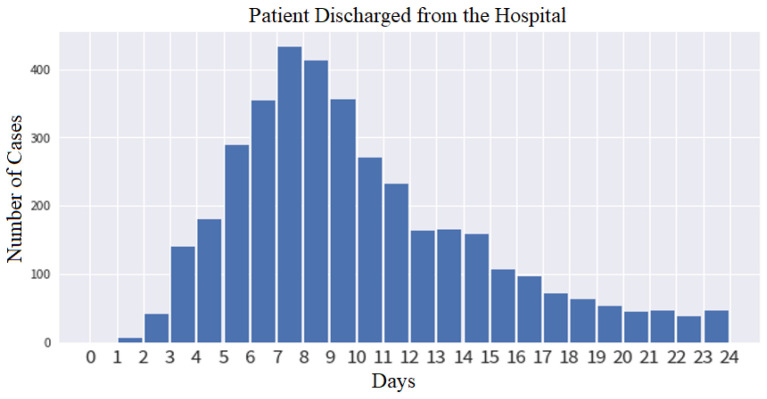
Data observations and statistics for the 24 days’ hospitalization.

**Figure 6:**
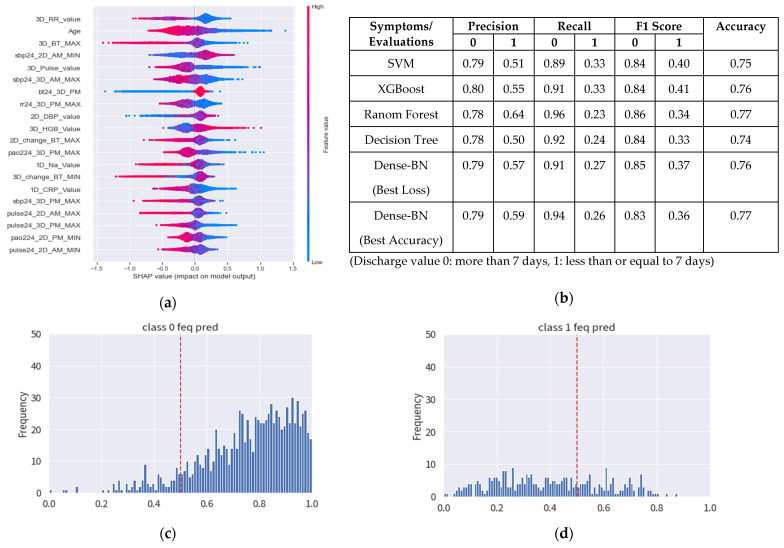
Feature importance and SHAP values: (**a**) SHAP based data analysis, (**b**) Machine and deep learning-based results, (**c**) No discharge confidence analysis for patients within 7 days, and (**d**) Discharge confidence analysis for the patients within 7 days.

The dataset used in this work belongs to a TCVGH, which has the observations and statistics as presented in Figure 5. The length of hospital days presents a maximum number of days of hospitalization (including emergency unit) as high as 104 days for the affected patients, whereas Figure 5 only presents for the 24 days, as it is found to be more effective having the x-axis as days and the y axis as number of cases. The features used within this experiment can be referred to in Table 4, whereas Table 7 provides detailed machine and deep learning results with cutoffs. In Figure 6, a new set of features are constructed by the addition of the new 12 h of features by the doctor’s recommendation to analyze the feature importance, SHAP scoring (Figure 6a), and applying it on machine and deep learning-based methods to have better prediction results (Figure 6b). The 12 h features include RR, Pulse, SBP, Pao2, and BT_metric for the evaluation. Figure 6c predicts the class 0 frequency as the no discharge prediction for the patients with high confidence, whereas Figure 6d shows the discharge prediction with less confidence. Therefore, it can be inferred that patients discharged within the hospital around 7 days may not have obvious clinical features within 3 days.

#### 3.1.2. CXR Imaging Sections for Symptom Categorization

The CXR image can be categorized based on the symptoms of infiltrate and cardiomegaly. Furthermore, the symptoms are also judged by the doctors based on the quality of the CXR, the locations of the symptoms observed, and the partitions into four equal CXR sections with the label severity. As given in Table 8, the labels were set to normal, slight, medium, and severe. Later, during the experiments in Table 9, the demonstration of the prediction accuracy was noticed quite higher for the normal and severe symptoms/labels. The infiltrate is given by four equal sections of the CXR and cardiomegaly as only independent CXR. The CXR images are processed in the following steps: (a) Establish an inference model for automatic grading of chest X-ray image infiltration, which is based on 600 doctors’ interpretation, (b) Image infiltration extraction feature by vector technology, and (c) Data and image integration for machine learning inference model establishment.

#### 3.1.3. Symptom Feature Extraction and CXR Combination with Vital Signs

**Figure 7:**
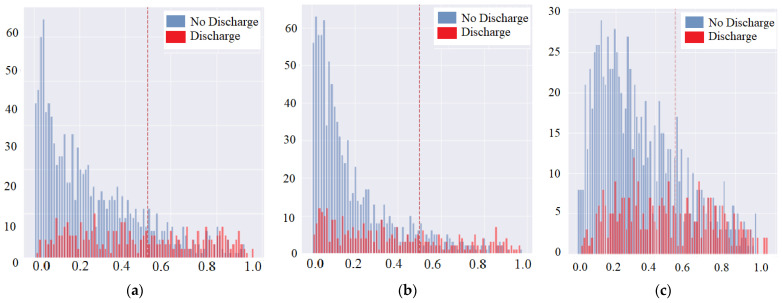
Performance Statistics for Class Weights: (**a**) Load Best 1 Weights, (**b**) Load Best 2 Weights, and (**c**) Load Best 3 Weights.

**Figure A1:**
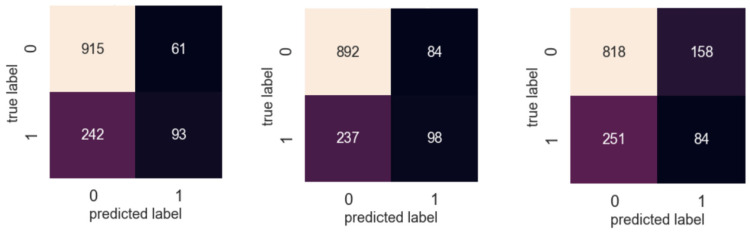
Confusion Matrix for Without Data Balancing Method and Different Best Weights.

**Figure A2:**
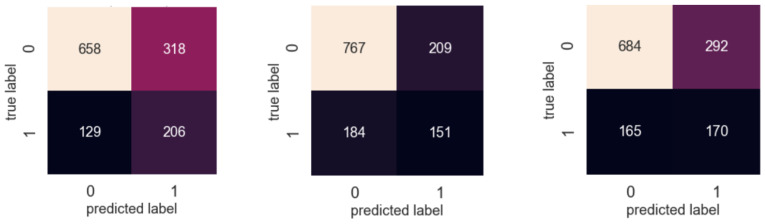
Confusion Matrix for Double Up Sampling Data Balancing Method and Different Best Weights.

**Figure A3:**
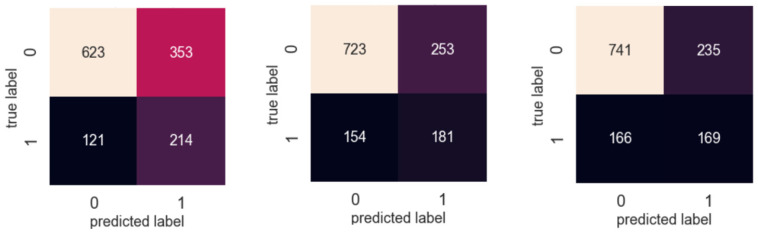
Confusion Matrix for Triple Up Sampling Data Balancing Method and Different Best Weights.

**Figure A4:**
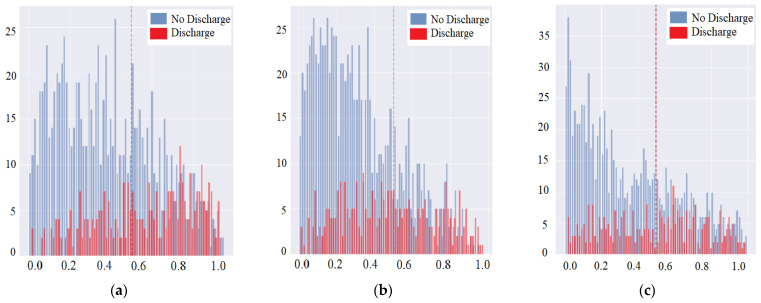
Performance Statistics for Double Up Sampling Method: (**a**) Load Best 1 Weights, (**b**) Load Best 2 Weights, and (**c**) Load Best 3 Weights.

**Figure A5:**
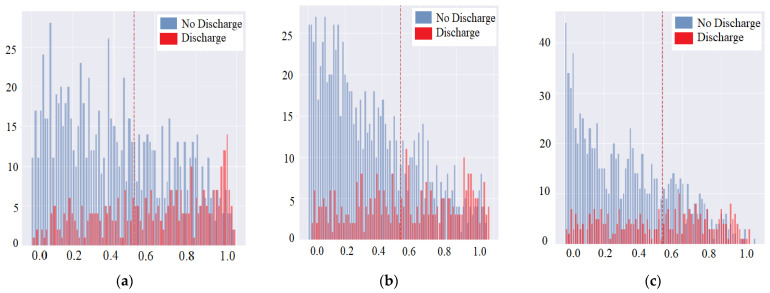
Performance Statistics for Triple Up Sampling Method: (**a**) Load Best 1 Weights, (**b**) Load Best 2 Weights, and (**c**) Load Best 3 Weights.

**Figure A6:**
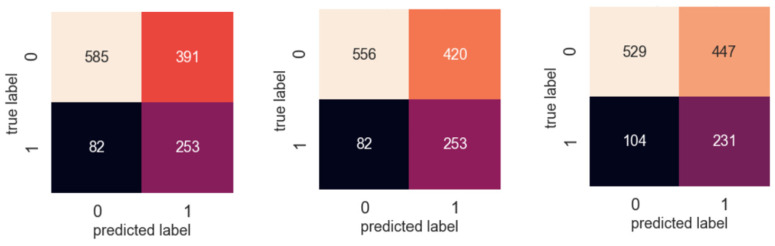
Confusion Matrix for Quadruple Up Sampling Data Balancing Method and Different Best Weights.

**Figure A7:**
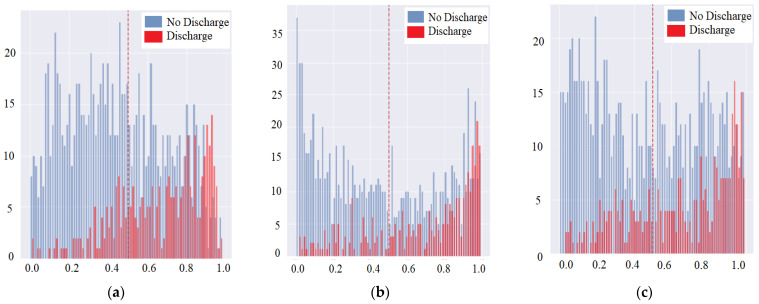
Performance Statistics for Quadruple Up Sampling Method: (**a**) Load Best 1 Weights, (**b**) Load Best 2 Weights, and (**c**) Load Best 3 Weights.

**Figure A8:**
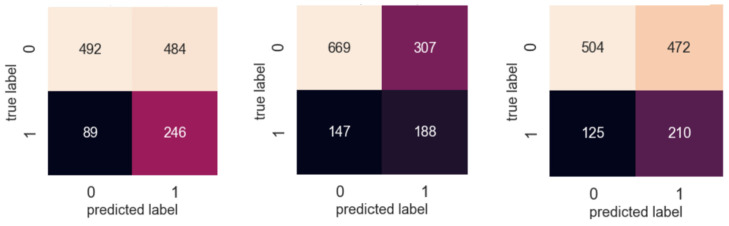
Confusion Matrix for Down Sampling Data Balancing Method and Different Best Weights.

**Figure A9:**
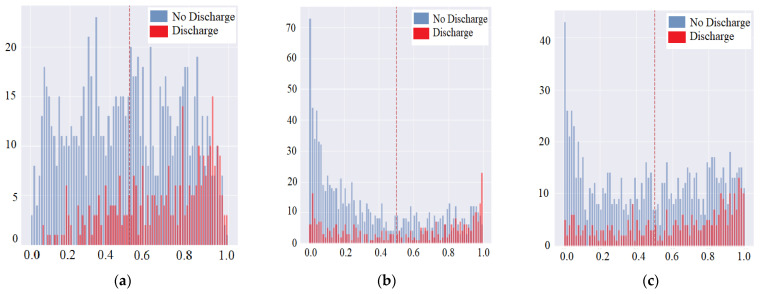
Performance Statistics for Quadruple Up Sampling Method: (**a**) Dense 1 Weights, (**b**) Dense 2 Weights, and (**c**) Dense 3 Weights.

**Figure A10:**
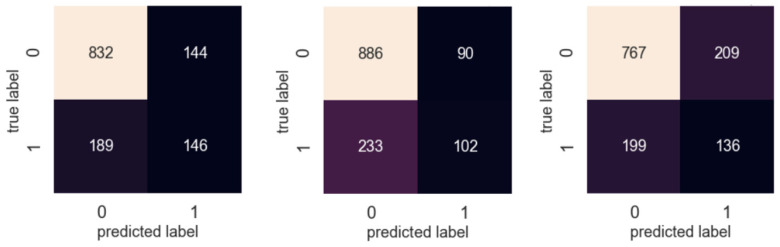
Confusion Matrix for Class Weights based Data Balancing Method and Different Best Weights.

The severity discrimination model of each affected area to extract feature vectors from the average of first and last available patients CXR images is used to predict discharge in this scenario. The combination of four parts of the infiltrate and the symptoms of cardiomegaly is to check for a possible improvement within the results. The interpretable layers added within the dense CNN are used to check which layer size performs best for the prediction improvement. Adding cardiac enlargement/cardiomegaly feature results helps to significantly improve the identification of the patients who can be discharged from the hospital within 7 days. These results are considered to be slightly better than using vital signs only, in comparison to the symptom feature extraction combined with the vital sign, i.e., average values of two X-ray from the first 3 days of the admittance in the hospital. Similarly combining the features of the infiltrate, cardiomegaly, and vital signs indicates that slight improvement with the constructed CNN layer can also provide a better output. Adding the vital signs and cardiac enlargement characteristics at the same time has limited the improvement in the identification of the patients, who can be discharged from the hospital within 7 days, whereas the significance of adding the heart enlargement feature and adding the vital sign feature may be very similar to those who can be discharged within 7 days. The data balancing methods are necessary for the unbalanced data to make the model training well. In Figure 7, the performance statistics for class weights is shown, which indicates the down sampling has a significant increase in label 0 in the original class weight experiment, and the label 1 is found to be declining. Even if having balanced data in both the discharge/no discharge categories, there is no improvement in the results. The details of the naïve, up sampling, down sampling, and class weight-based evaluation can be referred to in the Appendix A Section from Figure A1, Figure A2, Figure A3, Figure A4, Figure A5, Figure A6, Figure A7, Figure A8, Figure A9 and Figure A10 and Table A1, Table A2, Table A3 and Table A4.

Thus, an optimal cut point is selected based on the balance within the positive and negative predictions by avoiding the false alarm simultaneously. Therefore, the cut point is considered to be of high importance as it is critical to the MDA-PSP model success factor.

**Figure 8:**
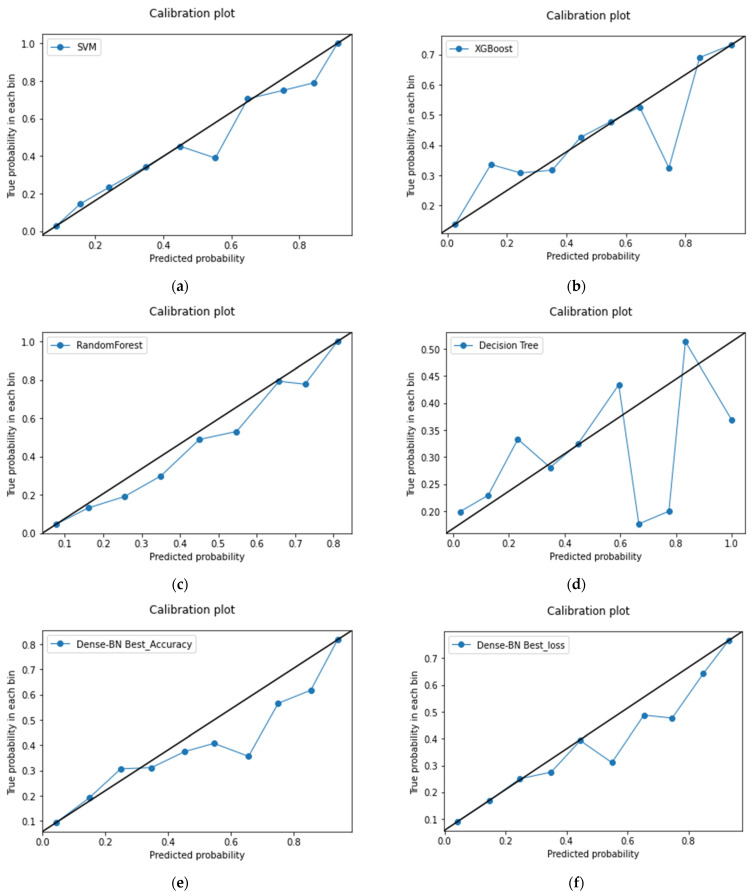
Calibration Plots for (**a**) SVM, (**b**) XGBoost, (**c**) random forest, (**d**) decision tree, (**e**) dense-BN accuracy and (**f**) dense-BN loss.

In the case of no class-weights used during the experiments, the F1 score for label 0 has improved but no significant improvement is noticed in label 1. Table 10 shows that in the case of dense layers with class weights, a slight decrease in label 0 is noticed but a significant improvement to 0.47 from 0.38 in comparison to no class weights. Figure 8 provides a calibration plot for MDA-PSP machines and deep learning algorithms for detailed analysis to evaluate how much the classifier is calibrated, i.e., every class label has differing probabilities that are measured. The linear straight line is the ideal calibrated model curve.

## 4. Discussion

The MDA-PSP overall findings are summarized as the combined CXR symptom vector and vital sign to build a model for discharge prediction, which have training accuracy of 75%, accuracy rate of not being discharged as 81%, and accuracy rate of discharge as 50%. In the discussion section, a detailed discussion about the various factors directly and indirectly affecting the patient status for the discharge is presented. Later, the Venn diagram will be presented to study logical relations between the sets for similarities and differences.

### 4.1. The following Factors Are Completely Variable and the Occurrence of It Depends on Health, Mental, Financial Status, etc.

- Insurance: In Taiwan, the government provides national health insurance (NHI) cards to the citizens. The NHI card requires a once a year payment of nominal amount, which then provides health insurance for any major natural or accidental treatment within any Taiwanese major hospital. Even though it provides insurance for any case, the time period for the patient to be admitted should be no more than 7 days for the refund. So, this forms the need for one of our motivations to design an XAI model for prediction of the patient’s discharge on the 7th day. Due to insurance benefit conditions, both the doctor and patient prefer to have discharge within 7 days of the hospital admittance.
- Suitable Discharge Time: The suitable discharge time for the patient is considered to be less than or equal to 7 days in the treatment. Nevertheless, less time is always favorable as it can save hospital resources as well as of the doctors, patients, family, hospital staff, etc. Whereas in the case of unfavorable cases, the patient is required to stay for a longer time duration that may lead to losing the insurance claim for the refund. The suitable discharge time can also refer to the minimum treatment time required based on the patient’s health severity and may be the trainee doctor’s decision. The pneumonia is known to happen in any age range from one day born infant to any adult patient, so the decision also considers the medicine treatment effects. In the case of diet based on certain regional, lifestyle factors, the discharge time may vary, too.
- Doctor’s Recommendation: In rare cases, the doctors usually recommend the patient to take a delayed discharge. Complexity of the case may depend on age, lifestyle for the slower recovery, or adapting to the normal health status. In addition, there are foreign patients who need to be treated exclusively and by experienced doctors, as the treatment approach and the recovery may vary depending on different continents. In some cases, the treatment by some medicine may react in the medical report. Therefore, the doctor needs more time to change the treatment and to have patience for the recovery process.
- Patient’s Mental Status: The discharge time also depends on the patient’s willingness or feeling energetic to confirm complete recovery. The doctors usually check for vital signs and medical reports for the discharge approval. In rare cases, if the patient is not mentally ready to discharge and possess good financial background, then the doctor may allow to continue stay depending upon beds availability. In some cases, if a patient is addicted to some habits, then he prefers to stay until complete recovery. It can also depend on how much the patient needs medical facilities to be received in a special exclusive room for the admittance. As soon as the patient is recovered, the doctor recommends discharge and continues to monitor vital signs remotely by using sensor watch, video camera-based consulting sessions, etc. The patient then later on can stay connected with the specialist doctor to report the symptoms, if any, as the follow-up.
- Re-admission Issue: In some exceptional circumstances [48,49], the patient has to go through the re-admission in the hospital. In the case of mid-size hospitals, there may be an undetected issue or the specialist doctor and medical condition predicting machines are not available in the emergency situation. Moreover, if the patient is in the transfer period because of his work, business, family shifting, or other factors, then the patient has to request for transfer options with the hospital by co-operation process. In some insurance cases, the patient can only get refund advantage of the hospital charges with referring to some specific hospital. Possibly, there can be some recommendations by the family or doctor to shift to a specialty hospital for fast recovery and experienced approach towards serious health conditions.
- Family Care/Support: In the case of some single people residing remotely [50] from the hometown region, the doctor may advise the patient to stay a couple of days more for the complete health recovery convenience. Even if there are some foreign patients working in the company, they may need special treatment and consulting for the health recovery. When the family and financial support is good, then the services can be shifted to the patient’s home by a visiting doctor. Whereas in the case of serious health conditions, the doctor may advise the patient to shift to a exclusive room, reserved for the special treatment facility.
- Multiple Disease Disorder (Comorbidity) of a Patient: In special cases, a patient is admitted in the hospital with a chronic disease [51]. Later on, a past or new disease is diagnosed, which is required to be treated carefully. In such cases, extra time is required for the complete health recovery of the patient. There are some cases when an addicted patient needs psychological counseling for controlling habits and adopting a healthy lifestyle. A complex case can be stated as when there is a dependency between the diseases, which may require specialist treatment. Even though it is a rare case to extend discharge time for the secondary disease, it needs to be cured. When a patient may have to stay for the secondary disease for more time than expected initially, the hospital must provide necessary support for such extension. Ultimately, it is recommended by the doctor to cure completely rather than schedule follow-up for the diagnosis and treatments. Pneumonia is the third of the top ten causes of death in Taiwan as well as in the world. The longer the hospital stay, the more likely it is to cause complications, hypoxemia, anemia, hypoalbuminemia, etc.
- Deploying AI System in the Hospital: Considering most of the application domain, AI models help to predict a patient’s conditions but not to diagnose the patient’s outcomes. Doctors are responsible for diagnosis and taking actions with treatments, and AI helps to provide decision choice and recommendations. AI models provide high-quality recommendations for junior doctors. Hands-on experiences are one of the major parts of doctor training. Young doctors will have a high-quality baseline of patient diagnosis with the help of AI models. Moreover, it improves the patient-care quality, even saving patient’s lives. In most hospitals, doctors are a critical resource and almost overrun. It is not possible to take care of all patients at any time. Automatic patient’s data collection for AI models will strongly help the patients care for 24 h/day. If the sensitivity and specificity of AI model predictions are good enough, it will greatly relieve the loading of doctors, and provide persistent health-care service during patient’s stay period in the hospital.

### 4.2. Venn Diagram Presentation for the Detail Analysis of the Prediction Results

**Figure 9:**
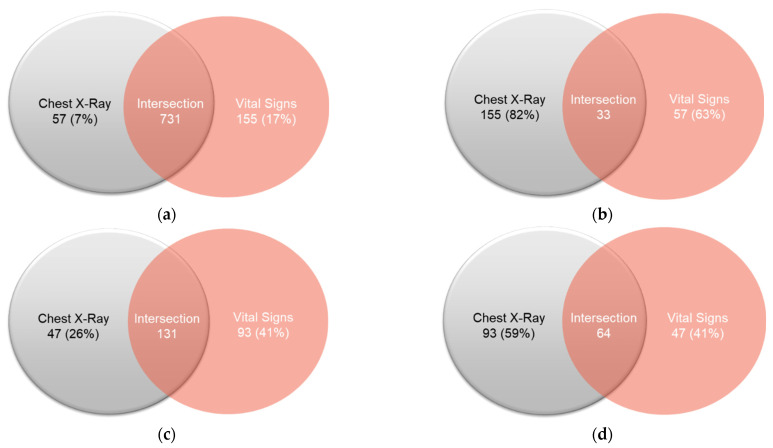
Intersection diagram for the patient symptoms-based prediction: (**a**) True Positive (TP), (**b**) False Positive (FP), (**c**) False Negative (FN), and (**d**) True Negative (TN).

The intersection diagram is used to observe the feature intersection of the vital signs and CXR for the prediction analysis. The four Venn diagrams are produced as given in Figure 9 and Table 11:

- Cannot be discharged within seven days, the prediction is correct (true positive).
- Unable to be discharged within seven days, the prediction was wrong (false positive).
- Able to be discharged within seven days, the prediction was wrong (false negative).
- Able to be discharged within seven days, the prediction is correct (true negative).

**Figure 10:**
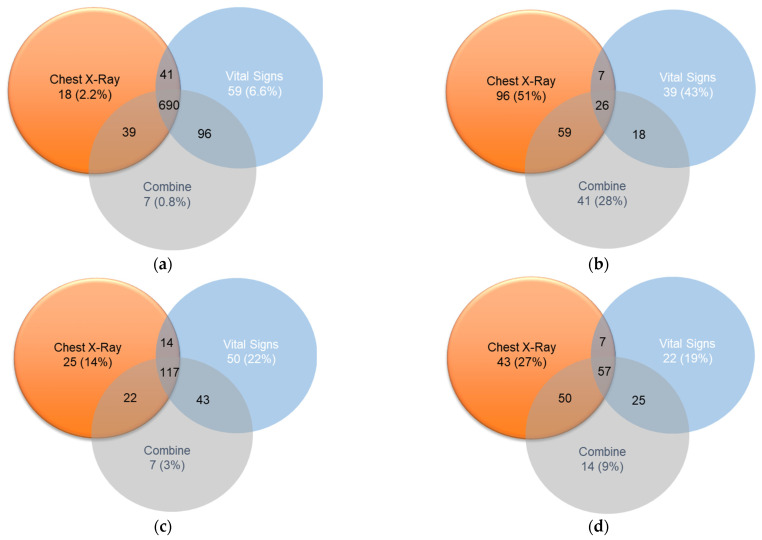
Venn diagram for the patient symptoms-based prediction and its intersection: (**a**) True Positive, (**b**) False Positive, (**c**) False Negative, and (**d**) True Negative.

The patient numbers are used in each set to calculate the average scores of the symptoms. Each set will be combined to calculate the scores that include four symptoms (four sections of lung infiltration) and seven symptoms (four types of lung infiltrate, cardiac hypertrophy, and two types of pulmonary hydrops). The label 0 outcome means hospitalization for more than seven days and label 1 outcome is the patient can be discharged within 7 days. Therefore, it is proved that for Figure 9a, vital signs are more accurate and for Figure 9d, CXR is more accurate. The Venn diagram proved that different data (CXR and Vital Sign) as shown in Figure 10 and detailed in Table 12 have high reproducibility for patients who can be discharged from the hospital (label 0), which means that these patients have characteristics leading to the success of both models. For patients who cannot be discharged from the hospital (label 1), the repeatability is significantly reduced. On the contrary, the part of mis-guessing label 1 is significantly improved. The uncertainty of the data representing label 1 is higher, which also confirms our past experiments; the predictive ability of label 1 is also relatively low.

### 4.3. Limitations of the Prediction System

The following are the limitations of the MDA-PSP system:

- The medical doctors need to be trained for interpreting classification results and identifying false positive cases.
- The classification model must adapt after appending new data for training and evaluation.
- The hidden layers in the dense do not provide complete information for the doctor’s detail analysis. In the future, the system can be made more transparent at every step using explainable AI (XAI).

## 5. Conclusions

Pneumonia is the third of the top ten causes of mortality in the world including Taiwan. To overcome such serious respiratory disease, MDA-PSP provides effective data preprocessing operations by SHAP feature analysis, imputation, adaptive imputation for vital signs, and CXR by using classification to achieve better outcome. The data preprocessing and class weights-based classification is the most prominent process for the evaluation. Therefore, for the patients who are admitted in the general ward because of pneumonia, their symptoms are evaluated to have prediction of discharge within 7 days. The dense-BN with class weights has provided accuracy of 75% by the multi-modal data analysis. Various methods from machine and deep learning is applied to have an overall analysis and their performance comparison prediction on the patient’s data. In the future, we plan to provide clinical care suggestions, assist doctors in decision-making, and control the number of beds, which can be extended to medical institutions with insufficient equipment. Additionally, we plan to have cross-academic model effectiveness verification and apply for Software as a Medical Device (SaMD). Also, need to design a plan on introducing clinical auxiliary care and observing the effectiveness of clinical application, such as hospitalization days, medical expenses, etc.

## Figures and Tables

**Table 1:** Comparison of Various Pneumonia Detection Models.

References	Study Aims	Source/ Input Data	Preprocessing/ Statistical Analysis	Machine Learning/Deep Learning Algorithms	Evaluation
D. Zeiberg et al. 2019 [22]	A machine learning approach to risk stratifying patients for ARDS.	Electronic Health Record (EHR)	5-fold cross validation and categorical data transforms.	Extreme gradient boosted (XGBoost) decision tree model.	AUROC
V. Chouhan et al. 2020 [23]	Pneumonia prediction using ensemble model.	CXR dataset, pre-trained on ImageNet.	Augmentation techniques: Random Horizontal Flip, Random Resized Crop, and Varying Intensity.	AlexNet, DenseNet121, ResNet18, InceptionV3, and GoogLeNet.	Area under the receiver operating characteristic curve (AUROC)
A. Yamagata et al. 2020 [24]	To investigate the prognostic factors related to 30-day mortality in patients.	Nursing and healthcare-associated pneumonia	Student’s t-test and chi-squared test	Univariate analysis and multivariate analysis using stepwise logistic regression.	*p* value
J. Zhang et al. 2020 [25]	A confidence aware anomaly detection (CAAD) model, with a shared feature extractor, an anomaly detection module, and a confidence prediction module for viral pneumonia.	CXR Dataset	Weights in ImageNet	CAAD model/Dense-Net and One-Class SVM (OC-SVM).	AUC, accuracy, sensitivity, and specificity.
MDA-PSP	Pneumonia status prediction for patient discharge within 7 days or not.	Vital Signs and CXR	Standard Scalar, SHAP, imputation, adaptive imputation, and SMOTE.	Dense-BN with class weights, dense layers, XGBoost, RF, SVM, and decision tree.	Precision, recall, F1 score, and accuracy.

**Table 2:** The Vital-Sign Demographic Details (showed the first three-day data collected between the year 2014 and 2018).

Serial No.	Vital Sign	Range (Total = 3972)	Mean (within 7 Days–after 7 Days)		Median (within 7 Days–after 7 Days)		Mode (within 7 Days–after 7 Days)	
1	Age	18–106	65.09	72.88	66	76	62	88
2	Gender (M:1)	64%	62%	65%	1	1	1	1
3	Glutamic-Oxalacetic Transaminase (GOT U/L)	6–1251	28.17	29.95	23	23	23	23
4	Glutamic-Pyruvic Transaminase (GPT U/L)	0–1488	29.77	30.36	22.5	22.5	30	30
5	White Blood Cell (WBC/μL)	80–70,630	10,425.45	11,536.66	9620	10,500	7250	7250
6	Hemoglobin (HGB g/dL)	4.6–20.9	12.23	11.48	12.5	11.4	14	11
7	Platelets (PLT × 1000/μL)	3–1066	228.36	237.42	217	220	275	275
8	Sodium (Na meq/L)	100–163	135.58	135	136	136	136	136
9	Potassium (K meq/L)	1.7–6.5	3.88	3.93	3.8	3.9	3.7	4
10	Blood Urea Nitrogen (BUN mg/dL)	3–167	21	25.43	16	19	15	15
11	Creatinine (Cr mg/dL)	0.05–19.03	1.39	1.47	0.98	1.05	1.05	1.05
12	C-Reactive Protein (CRP mg/dL)	0.004–52.16	7.85	9.41	5.78	7.28	0.15	0.15
13	Glucose (Glu mg/dL)	13–1048	131.62	147.24	114	123	95	95
14	Respiratory Rate (RR /min)							
	Day 1	17–100	20.45	21.75	20	20	20	20
	Day 2	14–120	20.24	21.65	20	20	20	20
	Day 3	12–116	19.87	21.53	20	20	20	20
15	Systolic Blood Pressure (SBP mmHg)							
	Day 1	74–220	126.37	127.31	124	125	110	109
	Day 2	73–222	127.79	128.65	125	127	117	118
	Day 3	75–229	127.90	129.83	127	128	121	122
16	Diastolic Blood Pressure (DBP mmHg)							
	Day 1	6–108	62.97	61.03	62	60	56	60
	Day 2	13–115	64.40	61.90	63	61	65	59
	Day 3	0–105	65.32	63.03	64	62	63	60
17	Pulse (/min)							
	Day 1	56–901	102.36	105.93	100	104	100	100
	Day 2	56–192	93.96	100.49	93	99	92	100
	Day 3	53–779	90.20	97.98	89	97	84	100
18	BT_MIN (min °C)							
	Day 1	30–38.8	36.43	36.39	36.4	36.4	36	36
	Day 2	33.1–38.4	36.22	36.26	36.2	36.2	36	36
	Day 3	33.9–37.8	36.11	36.19	36.1	36.2	36	36
19	BT_MAX (max °C)							
	Day 1	35.4–41.4	38.04	37.95	38	37.9	38.1	37.5
	Day 2	34.4–40.4	37.38	37.54	37.3	37.4	37.2	37.4
	Day 3	35.2–40.3	36.97	37.27	36.9	37.2	36.8	37.2

**Table 3:** Comorbidity Dataset Details.

Serial No.	Comorbidity	Mean (within 7 Days–after 7 Days)		Median (within 7 Days–after 7 Days)		Mode (within 7 Days–after 7 Days)	
1	Cancer	38.22%	41%	0	0	0	0
2	Cardiovascular Disorder	51.57%	65.27%	1	1	1	1
3	Neurological Disorder (Non-Stroke)	29.93%	40.78%	0	0	0	0
4	Neurological Disorder (Stroke)	16.59%	24.36%	0	0	0	0
5	Respiratory Disorder	59.87%	71.95%	1	1	1	1
6	Diabetes	22.59%	29.29%	0	0	0	0
7	Gastroenterology	43.85%	54.34%	0	1	0	1
8	Renal Disorder	33.75%	43%	0	0	0	0

[0: No Discharge, 1: Allow Discharge].

**Table 4:** Feature Table of the Categorized Vital Signs.

Clinical Feature Screening	Category	Parameters
Once in a Day (24 h).	3 days’ lab data and its differences.	PaO2, pH, HGB, HCT, WBC, CRP, PCT. Cr, BUN, PLT, Ca, GLU, ALB, K, NA, GPT, GOT. and AGE.
	Comorbidity with 0/1 value as categories	Sex, Cancer, Cardiovascular disorder, Neurological disorder (non-stroke), Neurological disorder (stroke), Respiratory disorder, Diabetes, Gastroenterology, and Renal disorder.
	Vital signs (3 days: 0/1 value)	RR, Pulse, SBP, DBP, and BT.
Twice in a Day (Every 12 h).	3 days’ score corresponding data.	RR, Pulse, SBP, PaO2, BT_MIN, and BT_MAX.
	Category data.	RR, Pulse, SBP, PaO2, and BT as metric.

[Highlights: Scope of Complications].

**Table 5:** Vital Sign for Checkup by the Physician with the Normal Cut-off.

Vital Sign	Cut-Off (Normal)
Age	≥20
2. Pulse Rate	<100/min
3. Oxygenation (SaO2)	≥95%
4. Respiratory Rate	≤24/min
5. Body Temperature	≤37.8 °C
6. SBP	>90 mmHg
7. Blood Oxygen Saturation	>90%

**Table 6:** System Configuration.

System	Workstation (Windows 10, 64-bit OS)
Processor	AMD Ryzen 9 3900XT @ 4.7 GHz
Memory	TridentZ RGB 32G, DDR4-3200
Graphics Card (GPU)	Gigabyte AORUS RTX2080Ti 11G
Python Library	Numpy, Pandas, Matplotlib, Seaborn, OpenCV, and Keras.

**Table 7:** Detailed Machine and Deep Learning Evaluation with Cutoffs.

Algorithms	Cut-off Threshold	Precision (0/1)	Sensitivity (95% CI)	Specificity (95% CI)	F1-Score (0/1)	Accuracy	PPV/NPV	AUROC (95% CI)
**SVM**	0.3	0.83/0.48	0.51 (0.46–0.57)	0.81 (0.78–0.83)	0.82/0.49	0.73	0.48/0.83	0.64–0.68
	0.5	0.79/0.58	0.27 (0.23–0.32)	0.93 (0.91–0.95)	0.85/0.37	0.76	0.58/0.79	0.58–0.63
	0.7	0.77/0.79	0.13 (0.1–0.17)	0.99 (0.98–0.99)	0.86/0.23	0.77	0.79/0.77	0.54–0.58
**XGBoost**	0.3	0.82/0.49	0.45 (0.4–0.5)	0.84 (0.81–0.86)	0.83/0.47	0.74	0.49/0.82	0.62–0.67
	0.5	0.8/0.55	0.33 (0.29–0.39)	0.91 (0.89–0.92)	0.85/0.42	0.76	0.55/0.8	0.6–0.64
	0.7	0.78/0.59	0.21 (0.17–0.26)	0.95 (0.93–0.96)	0.86/0.31	0.76	0.59/0.78	0.56–0.6
**Random Forest**	0.3	0.85/0.39	0.66 (0.61–0.71)	0.65 (0.62–0.68)	0.74/0.49	0.65	0.39/0.85	0.63–0.68
	0.5	0.78/0.59	0.21 (0.17–0.26)	0.95 (0.94–0.96)	0.86/0.31	0.76	0.59/0.78	0.56–0.6
	0.7	0.75/0.73	0.02 (0.01–0.05)	1 (0.99–1)	0.86/0.05	0.75	0.73/0.75	0.5–0.52
**Decision Tree**	0.3	0.78/0.36	0.39 (0.34–0.44)	0.77 (0.74–0.79)	0.77/0.37	0.67	0.36/0.78	0.55–0.6
	0.5	0.78/0.37	0.31 (0.26–0.36)	0.82 (0.79–0.84)	0.8/0.34	0.69	0.37/0.78	0.54–0.59
	0.7	0.78/0.39	0.3 (0.25–0.35)	0.84 (0.82–0.87)	0.81/0.34	0.7	0.39/0.78	0.55–0.59
**Dense-BN** **(Accuracy)**	0.3	0.84/0.43	0.61 (0.56–0.66)	0.72 (0.69–0.74)	0.78/0.5	0.69	0.43/0.84	0.64–0.69
	0.5	0.8/0.49	0.35 (0.3–0.4)	0.88 (0.86–0.9)	0.84/0.41	0.74	0.49/0.8	0.59–0.63
	0.7	0.77/0.64	0.17 (0.13–0.21)	0.97 (0.96–0.98)	0.86/0.27	0.76	0.64/0.77	0.55–0.59
**Dense-BN** **(Loss)**	0.3	0.85/0.41	0.66 (0.61–0.71)	0.67 (0.64–0.7)	0.75/0.51	0.67	0.41/0.85	0.64–0.69
	0.5	0.81/0.47	0.44 (0.39–0.49)	0.83 (0.8–0.85)	0.82/0.45	0.73	0.47/0.81	0.61–0.66
	0.7	0.78/0.58	0.23 (0.19–0.28)	0.94 (0.93–0.96)	0.85/0.33	0.76	0.58/0.78	0.57–0.61

**Table 8:** CXR Scoring Criteria for Feature Extraction.

Feature Category	Label
1. CXR	(Quality) 1: Good, 2: Medium, and 3: Bad.
2. Infiltrate (Location: 1: Top Left, 2: Bottom Left, 3: Top Right, and 4: Bottom Right)	(Symptoms) 0: Normal, 1: Slight, 2: Medium, and 3: Severe.
3. Cardiomegaly	(Symptoms) 0: Normal, 1: Slight, 2: Medium, and 3: Severe.

**Table 9:** Symptom Categorization Evaluation by DensNet-121 for infiltrates section-wise in four parts and independent cardiomegaly.

Symptoms/Evaluations	Precision		Recall		F1 Score		Accuracy
	0	1	0	1	0	1	
Infiltrate–1	0.91	0.63	0.84	0.75	0.87	0.68	0.82
Infiltrate–2	0.88	0.77	0.51	0.96	0.64	0.86	0.79
Infiltrate–3	0.91	0.51	0.88	0.58	0.89	0.55	0.82
Infiltrate–4	0.77	0.79	0.85	0.69	0.81	0.74	0.78
Cardiomegaly	0.96	0.71	0.94	0.81	0.95	0.76	0.92

(Discharge value 0: more than 7 days, 1: less than or equal to 7 days).

**Table 10:** Performance Evaluation for the Class weights and Dense Layer Comparison.

Symptoms/Evaluations	Precision		Recall		F1 Score		Accuracy
	0	1	0	1	0	1	
4 infiltrates with cardiomegaly by class weight [0.68335901, 1.86344538]	0.81	0.50	0.85	0.44	0.83	0.47	0.75
4 infiltrates with cardiomegaly by no class weights	0.79	0.60	0.94	0.28	0.86	0.38	0.77

(Discharge value 0: more than 7 days, 1: less than or equal to 7 days).

**Table 11:** Feature intersection for the CXR and Vital Signs analysis for the average scores.

Confusion Matrix / Evaluations	Chest X-ray (A)	Vital Sign (B)	Combine Image/Feature Points					
			A		B		A ⋂ B	
			4 Features	7 Features	4 Features	7 Features	4 Features	7 Features
TP	788	886	4.754	5.421	4.384	4.881	5.32	6.549
FP	188	90	4.384	4.881	4.754	5.421	3.985	4.303
FN	178	224	4.564	5.202	5.141	6.328	3.763	4.226
TN	157	111	3.763	4.226	4.564	5.202	3.391	3.57

**Table 12:** Venn intersection for the CXR, Vital Signs, and Combined result analysis for the average scores. (F-Features).

Confusion Matrix/ Evaluations	Chest X-ray (A)	Vital Sign (B)	Combine (C)	Combine Image/Feature Points													
				A		B		C		A ⋂ B		B ⋂ C		A ⋂ C		A ⋂ B ⋂ C	
				4 F	7 F	4 F	7 F	4 F	7 F	4 F	7 F	4 F	7 F	4 F	7 F	4 F	7 F
TP	788	886	832	4.22	4.56	3.98	4.37	4.07	4.71	4.51	5.23	4.63	5.19	5.0	5.82	5.37	6.63
FP	188	90	144	4.63	5.19	5.0	5.82	4.51	5.23	4.07	4.71	4.22	4.56	3.98	4.37	3.96	4.19
FN	178	224	189	4.36	4.88	3.57	3.84	3.64	3.93	4.29	5.47	3.99	4.67	4.79	5.57	5.24	6.43
TN	157	111	146	3.99	4.67	4.79	5.57	4.29	5.46	3.64	3.93	4.36	4.88	3.57	3.84	3.36	3.53

## Data Availability

The private data is not allowed to be disclosed due to hospital policy.

## Abbreviations

Auto-encoder (AE); Artificial Intelligence (AI); Acute Kidney Injury (AKI); Acute respiratory disease syndrome (ARDS); American Thoracic Society (ATS); Amazon Web Services (AWS); Batch Normalization (BN); Confidence aware anomaly detection (CAAD); Community-acquired pneumonia (CAP); Convolutional Neural Filter (CNF); Chest X-ray (CXR); Confusion, Urea nitrogen, Respiratory rate, Blood pressure (CURB-65) for age 65 and older.; Electronic Health Record (EHR); Extremely Random Trees Classifier (ERTC); Extreme gradient boosted (XGBoost); Gradient Boosted Classifier (GBC); Intensive Care Unit (ICU); Infectious Diseases Society of America (IDSA); Machine Learning (ML); Mean squared error (MSE); Multi-Layer Perceptron (MLP); National Health Insurance (NHI); One-Class Support Vector Machine (OC-SVM); SHapley Additive explanation (SHAP); Standard Operating Procedure (SOP); Software as a Medical Device (SaMD); Synthetic Minority Oversampling Technique (SMOTE).

## Appendix A

The appendix section presents supplementary data which will help to support additional details of the experiments results carried out in this work.

Table A1 shows raw vital signs data used without any preprocessing to be evaluated with the machine learning algorithm. It can be noticed that the F1 score is quite suffering in the evaluation. In the case of preprocessing used for data clean up as presented in Table A2 for hybrid dataset evaluation with different machine and deep learning-based methods, there is a significant improvement in the F1 score.

**Table A1:** Vital sign raw data evaluation on machine learning methods.

Machine Learning Algorithm/Evaluations	Sensitivity	Specificity	Accuracy	F1-Score
Logistic Regression	0.65	0.72	0.70	0.54
SVM	0.70	0.68	0.69	0.54
Gaussian NB	0.54	0.75	0.70	0.48
XGBoost	0.40	0.90	0.76	0.47

**Table A2:** Hybrid dataset evaluation after cleaned up with some popular machine and deep learning methods.

Machine Learning Algorithm/Evaluations	Dataset	Sensitivity	Specificity	Accuracy	F1-Score
Random Forest	Vital Sign	0.88	0.40	0.69	0.69
XGBoost	Vital Sign	0.91	0.33	0.76	0.74
Feature Extraction and DNN	CXR	0.81	0.47	0.72	0.72
Feature Extraction and DNN	CXR and Vital Sign	0.85	0.44	0.75	0.74

As discussed earlier, in the results as given below about the four-class and two-class based evaluation, the two-class evaluation emerges to be more accurate, and its detail output is shared in Table A3 and Table A4. The accuracy can be compared for Table A3 and Table A4 based on the same features by different class results. The confusion matrix and performance statistics for different method analysis is presented by Figure A1, Figure A2, Figure A3, Figure A4, Figure A5, Figure A6, Figure A7, Figure A8, Figure A9 and Figure A10. It shows that double up sampling in comparison to the null class weights has decline in label 0 but no significant improvement is noticed in label 1. Similarly, in the case of triple up sampling in comparison to the null class weights, it has slight decline in label 0 but no significant improvement is noticed in label 1.

**Table A3:** Chest X-ray Results (4-Class) based on the Neural Network Model.

Features/Evaluation	Precision	Recall	F1-Score	Accuracy
Infiltrates-Top Left	0.58	0.59	0.57	0.59
Infiltrates-Bottom Left	0.63	0.58	0.57	0.58
Infiltrates-Top Right	0.66	0.67	0.66	0.67
Infiltrates-Bottom Right	0.66	0.67	0.66	0.67
Cardiomegaly	0.71	0.72	0.71	0.72
Effusion Left	0.81	0.81	0.80	0.81
Effusion Right	0.78	0.72	0.74	0.72

**Table A4:** Chest X-ray Results (2-Class) based on the Neural Network Model.

Features/Evaluation	Precision		Recall		F1 Score		Accuracy
	0	1	0	1	0	1	
Infiltrates-Top Left	0.91	0.63	0.84	0.75	0.87	0.68	0.82
Infiltrates-Bottom Left	0.88	0.77	0.51	0.96	0.64	0.86	0.79
Infiltrates-Top Right	0.91	0.51	0.88	0.58	0.89	0.55	0.82
Infiltrates-Bottom Right	0.77	0.79	0.85	0.69	0.81	0.74	0.78
Cardiomegaly	0.96	0.71	0.94	0.81	0.95	0.76	0.92
Effusion Left	0.94	0.73	0.98	0.42	0.96	0.53	0.93
Effusion Right	0.95	0.43	0.98	0.23	0.96	0.30	0.93

Nevertheless, quadruple up sampling in comparison to the null class weights has decline in label 0 but a slight improvement is noticed in label 1. Ultimately, down sampling with no class weights is not considered as a good method for this experiment. Similarly, double and triple up sampling is not recommended. Whereas for quadruple up sampling, although label 1 has improved, the sacrifice is not good to practice in actual situations and test concentration.

## Appendix B

### Appendix B.1. Introduction (Extension)

AI-based status prediction is considered to be a better alternative [21], for the case of pneumonia as well as hospital planning for general ward/ICU space. In hospital management, the quality measure can be ensured by the MDA-PSP system as a part of product prototype development, preliminary verifications, and later used in real environment.

The background for MDA-PSP can be presented as standard scalar, synthetic minority oversampling technique (SMOTE), random forest, machine learning, deep learning, etc. [22]. Standard scalar is used to scale the data to a standard range. Each input variable is scaled separately by subtracting the mean then dividing by standard deviation for shifting the distribution to get the mean as zero and the standard deviation as one for dataset preprocessing. SMOTE is one of the statistical techniques to balance the number of cases in the dataset. So, the minority cases are handled by generation of new instances within the dataset, whereas the majority cases are unchanged, thus solving the imbalance issue within the dataset. The random forest classifier is a well-known meta estimator that is used to fit multiple decision tree classifiers on dataset subsamples. Averaging is used for the prediction accuracy improvement and over-fitting is kept in control. Machine learning is a subset of AI, which is used to make predictions based on training data. Most of the machine learning algorithms are based on statistical process analysis. It can be further categorized as supervised, unsupervised, and reinforcement learning. Deep learning is a well-known machine learning algorithm class that helps to construct and utilize multiple layers for different feature extraction from the input data. It is widely used in the image processing field for its application and classification.

In short, MDA-PSP uses a hybrid approach for predicting the status of pneumonia and improving the classification process. Ultimately, a good prediction and complete process is accomplished. In MDA-PSP, the research gap is concerned about multi-modal data analysis, which is quite scarce. This research is exclusively designed for demonstration of prediction on the 7th day of discharge status. The MDA-PSP research is rational as it provides prediction on the 7th day of discharge of patients affected by pneumonia using a hybrid approach. This approach is approved by the medical practitioners as the complete process for the assessment of the pneumonia disease as presented in the results of Section 4.

### Appendix B.2. Literature Survey (Extension)

Ultimately, the achievements of MDA-PSP can be summarized based on shortcomings analyzed from the above literature survey:

1. MDA-PSP uses both machine and deep learning-based algorithm to decide which approach evaluates to be effective for pneumonia prediction of the patient.
2. The use of multi-modal data analysis is considered to be efficient, which was less evaluated previously.
3. Mean-time analysis helps to provide recovery status within the 7th day treatment of the patient.
4. The properly defined pseudo-code of the algorithms and experiments will be helpful for detailed understanding and future research, and to simulate physician diagnosis and treatment, combine images and data for machine learning, and build predictive models.

### Appendix B.3. Experiments (Extension)

The precision, recall, F1 score, and accuracy are used to assess the performance of the system model and different machine and deep learning algorithms as given in equation w, x, y, and z, respectively:

(A1) $$\mathrm{Precision}=\frac{T_{P}}{T_{P}+F_{P}}$$

(A2) $$\mathrm{Recall}=\frac{T_{P}}{T_{P}+F_{N}}$$

(A3) $$F1\;\mathrm{Score}=\frac{2\times Precision\times Recall}{Precision+Recall}$$

where true positives ($T_{P}$) assures the patient status is infected and the model prediction was correct. True negative $\left(T_{N}\right)$ assures the patient is healthy and the model prediction was correct. False positives (*F_P_*) predict the model outcome as healthy whereas it is an error. False negatives (*F_N_*) predict the patient is healthy but actually is infected. The precision can be defined as classifier ability for not labelling negative samples as positive. The recall is also known as the classifier ability to detect positive samples. F1 score takes precision and recall as the harmonic mean for the output, whereas the model performance is measured using accuracy for the actual true positive from the overall positive predictions.

(A4) $$\mathrm{Accuracy}=\frac{T_{P}+T_{N}}{T_{P}+F_{N}+F_{P}+T_{N}}$$

(A5) $$\mathrm{Specificity}=\frac{T_{N}}{T_{N}+F_{P}}$$

Sensitivity also known as true positive rate is evaluated the same as the recall. Specificity is also known as true negative rate is inversely proportional to sensitivity. So, the increase in either one will decrease the other. Negative Predictive Value (NPV)/Positive Predictive Value (PPV) are known as clinical relevance of test. NPV/PPV specifies the test diagnosis for likelihood of a specific infection based on the prevalence of a condition. AUC-ROC is used to measure performance of the model classification based on different threshold values. AUC is defined as the measure of separability and ROC as the probability curve. The models distinguishing capability for infected and non-infected/healthy is evaluated using AUROC.
